# Evaluating participants’ satisfaction with supportive text message interventions during the 2023 wildfires and the impact of age and gender on overall satisfaction scores

**DOI:** 10.3389/fpubh.2026.1949324

**Published:** 2026-10-01

**Authors:** Gloria Obuobi-Donkor, Reham Shalaby, Belinda Agyapong, Ejemai Eboreime, Lori Wozney, Vincent I. O. Agyapong

**Affiliations:** 1 Department of Psychiatry, Faculty of Medicine, Dalhousie University, Halifax, NS, Canada; 2 Department of Psychiatry, Faculty of Medicine and Dentistry, University of Alberta, Edmonton, AB, Canada; 3 Mental Health and Addictions Program, IWK Health, Halifax, NS, Canada

**Keywords:** Alberta, Nova Scotia, satisfaction, text message intervention, wildfire

## Abstract

**Background:**

Supportive text message interventions provide reassurance and psychological support during high-stress situations like wildfires. By assessing user satisfaction with these interventions, we can gauge their effectiveness.

**Objective:**

This study explored subscriber satisfaction, experience, and perception related to the Text4Hope-AB and Text4Hope-NS text message interventions designed to provide psychological support during wildfires. It also explored the age and gender differences in overall satisfaction with the programs.

**Methods:**

A mixed-methods approach was utilized, involving both quantitative and qualitative data collection. Quantitative data were gathered using categorical responses to an online satisfaction and user experience survey, while qualitative data were collected through written responses to open-ended questions on the online survey. Participants included 312 individuals who received daily text messages over 3 months. An Analysis of Variance and Games-Howell *post hoc* analysis were used to examine age- and gender-related differences in overall satisfaction scores measured on a scale from 0 to 10.

**Results:**

The mean satisfaction score was 8.66 (SD = 1.63)/10. Most participants reported that the daily messages helped them cope with stress (76.0%), anxiety (73.4%), and depression (67.0%). In addition, 79.5% reported feeling connected to a support system, 77.9% felt hopeful about managing their mental health or addiction issues, 78.2% reported improved overall mental wellbeing, and 72.8% reported an enhanced quality of life. Participants generally perceived the messages positively. The qualitative analysis identified two main themes reflecting participants’ experiences and perceptions: expectations and recommendations, and experiences with the service. Satisfaction differed significantly by age and gender, with older participants generally reporting higher satisfaction than younger participants; male and female participants reported higher satisfaction than those identifying as other gender; however, the other-gender subgroup comprised only two participants, and this comparison should therefore be interpreted with considerable caution.

**Conclusion:**

Text4Hope-AB/NS were well received and positively perceived as useful and supportive during the wildfires. The findings demonstrate high user satisfaction, positive participant experiences, and differences in satisfaction across demographic groups. Future interventions may benefit from greater personalization and adaptation to the needs and preferences of different populations. Gender-related findings should be interpreted cautiously due to the small sample size.

## Introduction

Wildfires usually cause individuals to face heightened stress, anxiety, and uncertainty, which can significantly impact their mental wellbeing (1–3). For example, the one-month prevalence of depression, anxiety, and Post-Traumatic Stress Disorder during the Fort McMurray 2016 wildfires was 18.3, 15.7, and 10.2%, respectively (4). Individual perception and receptiveness to supportive text interventions during disasters are crucial to their effectiveness. Supportive text intervention programs have been increasingly recognized for their prospects of providing timely and accessible support to individuals during traumatic conditions (5, 6). They are valuable tools for delivering various health interventions, such as behavioral changes and psychological support after traumatic events (7–9). Literature has demonstrated that text message interventions are acceptable across diverse populations and bridge geographical barriers (10–12) with reports of increased hopefulness, feelings of being in control of managing depression and anxiety, and improved overall mental wellbeing (13, 14).

Supportive text messages can positively impact user satisfaction and mental health outcomes. For example, studies evaluating programs like Text4Mood, Text4PTSI, and MoreGoodDays have reported that subscribers felt more hopeful, in control of their mental health issues, and connected to a support system through supportive text message interventions (14–16). Additionally, supportive text messaging has been associated with reducing depressive symptoms, increasing abstinence duration in alcohol use disorder, and garnering high user satisfaction levels (13, 17). A study conducted in the Republic of Ireland reported that 83% of patients indicated that the text message intervention was beneficial in enhancing their mental health, specifically by serving as a motivator for recovery and helping to prevent relapse (13).

Despite their limitations in providing sophisticated interventions, research has also indicated that text messaging interventions offer significant opportunities to assess and address post-traumatic symptoms and related problems following war and disaster situations, like wildfires (18). For instance, after receiving text message intervention, participants’ mental wellbeing improved by 24.7% during the wildfire season in Alberta and Nova Scotia (6).

Supportive text messaging interventions may be particularly suitable during natural disasters, such as wildfires, because they are scalable, low-cost, accessible, and capable of reaching large populations rapidly, including individuals in remote or disrupted environments where traditional mental health services may be limited (15, 19). Text messaging also allows for timely psychological support without requiring internet access, in-person attendance, or extensive technological literacy, making it an important intervention during emergencies (20).

User satisfaction with supportive text messaging programs has been high, with over 80% of subscribers reporting improvements in their mental health in various surveys (21). These programs are feasible and acceptable ways of providing psychological interventions to individuals with mental health issues (15). Additionally, maximizing personalization and relevance in text message support has been found to increase the value of interventions and reduce the risk of disengagement (22). Text messaging interventions’ usability and user experience have been positively assessed, suggesting a positive reception among users in the general population (16) while wildfires are unknown.

Previous studies suggest that demographic factors such as age and gender may influence engagement, usability, and satisfaction with digital mental health interventions (23). Older adults may prefer simpler and more accessible technologies, such as text messaging, whereas younger adults may prefer more interactive digital platforms (24). Again, gender differences in experiences of social support and mental health interventions have been reported in the literature (25, 26). Assessing whether supportive text messaging interventions are perceived differently across demographic groups may help identify populations for whom these interventions are especially acceptable or may require adaptation.

One crucial factor affecting the acceptance of text message interventions is tailoring messages to the individual’s needs. Hence, text message intervention programs (Text4Hope-AB and Text4Hope-NS) were implemented in Nova Scotia and Alberta, Canada, during the 2023 wildfires to support individuals’ mental health. We hypothesize that most participants would perceive the Text4Hope-AB and Text4Hope-NS supportive text messaging programs as helpful and beneficial to their mental wellbeing, and that satisfaction may vary across demographic groups, particularly by age and gender, given evidence that these characteristics are associated with differences in engagement and adherence to digital mental health interventions (27, 28).

In this study, we evaluate end-users’ satisfaction with the programs in improving their mental wellbeing. The study examined the general experiences and perceptions of the Text4Hope-AB and Text4Hope-NS programs among participants and explored satisfaction differences by age and gender.

## Methods

### Study setting and design

The study employed a mixed design (qualitative and quantitative study designs). The voluntary response sampling approach was adopted. The study was conducted in Alberta and Nova Scotia, Canada, which were severely affected by devastating wildfires in 2023. In late April 2023, Alberta experienced a fire outbreak that ravaged nearly 2.2 million hectares between March and October 2023 (29). On the other hand, Nova Scotia experienced its largest recorded wildfire in history in May 2023 (30). The Text4Hope-NS and Text4Hope-AB programs were implemented as an emergency psychological response during the wildfire period.

### Data collection and program intervention

The data collection was done between 14 May and 23 September 2023. Residents of Alberta and Nova Scotia, Canada, completed a self-assessed online questionnaire through the Research Electronic Data Capture (REDCap) software. Participants self-subscribed to the program by texting “HopeNS” or “HopeAB” for residents in Nova Scotia and Alberta, respectively, to a short code number. Participants receive daily unidirectional supportive text messages for 3 months. These messages were developed from the cognitive behavioral therapy framework, created by mental health professionals with expertise in CBT, crisis intervention, and end-user engagement. They were designed according to CBT principles to provide emotional reassurance, coping encouragement, resilience-focused support, and normalization of stress responses during the wildfire crisis. All messages underwent structured clinical review to ensure clarity, appropriateness, and alignment with the Text4Hope implementation procedures. Intervention fidelity was maintained through a standardized, pre-specified delivery schedule with uniform message distribution and no modification during implementation. The supportive text messaging intervention was designed as an adjunctive source of support, not a sole source of psychological intervention. Participants continued to access their usual sources of support. As the intervention was implemented in a real-world setting, exposure to other sources of information could not be restricted or fully controlled. Examples of the supportive messages are:

*“No matter what setbacks you have faced or challenges that lie ahead, you can succeed if you have inner strength and stay focused. Have faith in yourself, and success will be yours no matter what problems the wildfire throws at you.”* (6).

*“There are 2 days in the week we should not worry about: yesterday and tomorrow. That leaves today, live for today. Thinking of the past or the future can be overwhelming for anyone facing a challenging situation or crisis.”* (6).

The messages were uploaded to an online platform that sent out automatic messages at 9 a.m. Mountain Time daily. The introductory message welcomed subscribers to the program and invited them to participate in an online survey. The questionnaires included demographic and user satisfaction surveys adopted from previous versions of Text4Mood satisfaction questionnaires (15) at 6 weeks and 3 months after enrollment. Participation in the program was voluntary, and intervention was offered without incentives. Receiving supportive messages was not contingent upon survey completion. Participants could opt out of the program anytime by texting “STOP.”

### 2.3 Outcome measure

The primary outcome measure was participants’ overall satisfaction with the Text4Hope-NS and Text4Hope-AB supportive text messaging programs and the age and gender differences in overall satisfaction. Overall satisfaction was assessed using participants’ responses on an 11-point Likert scale (0 = very dissatisfied, 5 = neutral, 10 = very satisfied).

The secondary outcome measure was the program’s impact on participants’ mental health and program feedback 6 weeks and 3 months after subscribing. Participants provided feedback on the text messaging intervention by responding to questions that focused on these areas:

a) Perception of how participants coped with some mental health symptoms (stress, anxiety, and depression), feel connected to a support system, perceive quality of life, and the impact on their overall mental wellbeing. These questions were measured on how they agreed, strongly agreed, neutral, disagreed, and strongly disagreed. During analyses, these responses were categorized into three (agree or strongly agree, neutral, and disagree or strongly disagree).
b) Feedback on the programs was measured on a 5-point Likert scale: always, often, sometimes, rarely, and never.
c) Open-ended questions to know how participants perceive the program and how to improve included:

“How can we improve Text4Hope-NS or Text4Hope-AB?”

“Please share additional comments, suggestions, or feedback about Text4Hope-NS or Text4Hope-AB.”

The validity and reliability of the survey items used to assess satisfaction have not been formally established. However, the items were informed by questions previously used to evaluate user satisfaction with similar supportive text messaging interventions (15, 16, 29). The items were selected based on their relevance to the objectives of the Text4Hope program and were reviewed by experts for clarity, relevance, and appropriateness for assessing participants’ experiences and perceptions of the intervention. These items were selected to assess participants’ perceived satisfaction with the intervention and its usefulness as an additional source of support (15, 16, 31).

### Sample size consideration

Given a population of 1,802 subscribers, assuming a 95% confidence level, a margin of error of 1 point, and a population variance of 100, the required sample size needed to estimate the overall mean satisfaction is 317 participants.

### Triangulation of the quantitative and qualitative analysis

#### Quantitative data

The data were analyzed using SPSS for Windows version 28 (IBM Corporation, Armonk, NY, USA) (32). Demographic characteristics were examined and were summarized as raw numbers and percentages. We employed a one-way analysis of variance (ANOVA) with two-tailed significance (*p*-value ≤ 0.05) to evaluate the differences in the participants’ satisfaction mean scores across their demographic characteristics, specifically age and gender, to compare participants’ level of satisfaction with the program. Tukey *post hoc* analysis was used for *post hoc* analysis; Welch’s *F* test and Games-Howell *post hoc* test were employed when the homogeneity of variance was violated. For the purposes of the ANOVA analysis, the effect size was computed using partial eta squared (sum of squares across groups/Total sum of squares), and when applying Welch’s *F* test, an adjusted omega squared formula (dfbet (*F* − 1)/dfbet (*F* − 1 + NT)). No imputation was performed for missing data, and the results were based solely on completed survey responses.

#### Qualitative data

Study participants were asked to share their comments and suggestions about the program. For qualitative data analysis, thematic analysis as described by Braun and Clarke (25) was utilized. Two researchers independently examined the participants’ responses without the assistance of any automatic coding software. Responses were re-read multiple times in order to become familiar with them as well as get the preliminary impression of what the participants had to say. Codes were produced by applying an inductive approach where significant text passages were systematically labeled according to the properties they possessed. Coding discrepancies were resolved through discussion and consensus. The codes were then organized into more general themes based on similarities and differences in responses received. The themes produced were discussed and fine-tuned among the research team. Sub-themes were derived from the main themes. Reflexive discussions were also held during the analysis stage. Finally, clear definitions were provided for each of the final themes. Data saturation was considered reached when no additional themes or subthemes emerged from the analysis. Verbatim participant quotations were used to illustrate and support each identified theme and subtheme.

Of the 1,802 participants who voluntarily subscribed to the program, 312 completed the satisfaction survey and were included in the final analysis, yielding an approximate 17.3% completion rate. The remaining subscribers did not provide a completed satisfaction survey and were therefore not included in the analysis.

## Results

### Quantitative data

Table 1 illustrates the demographic characteristics of the study participants. Middle-aged adults, 31–50 years old, predominated the population (105, 33.7%). Most participants were female (270, 86.5%) and Caucasian (261, 83.7%). Two hundred fifty-seven of the participants, comprising 82.4%, had completed post-secondary education. Their relationship status was primarily married, common law, or partnered (170, 56.9%), employed (198, 65.8%), and 203 (67.4%) owned their house.

**Table 1:** Demographic characteristics of participants.

Demographic characteristic	Total participants *n* (%)
Age (years)	
≤30	30 (9.6)
31–50	105 (33.7)
51–65	136 (43.6)
>65	41 (13.1)
Gender	
Male	40 (12.8)
Female	270 (86.5)
Other gender	2 (0.6)
Ethnicity	
Caucasian	261 (83.7)
Indigenous	16 (5.1)
Asia	17 (5.4)
Black	8 (2.6)
Other	10 (3.2)
Educational status	
Less than high school	4 (1.3)
High school	51 (16.3)
Post-secondary	257 (82.4)
Relationship status	
Married, common law, or partnered	170 (56.9)
Single	70 (23.4)
Separated or divorced	44 (14.7)
Widowed	15 (5.0)
Employment status	
Employed	198 (65.8)
Unemployed	43 (14.3)
Retired	48 (15.9)
Student	12 (4.0)
Housing status	
Own house	203 (67.4)
Rented accommodation	76 (25.2)
Live with family and friends	22 (7.3)

The results in Table 2 show that over half of the participants agreed or strongly agreed that the daily text messages helped them manage stress (76.0%), anxiety (73.4%), depression (67.0%), and loneliness (59.3%). Additionally, 79.5% felt that the messages fostered a connection to a support system, 77.9% felt hopeful about managing mental health or addiction issues, 78.2% reported improved overall mental wellbeing, and 72.8% experienced an enhanced quality of life.

**Table 2:** Differences in how daily messages are perceived based on gender.

Perceived Impact	Male *n* (%)	Female *n* (%)	Other gender *n* (%)	Total *n* (%)	*p*-value
Helped cope with stress					
Agree/strongly agree	31 (77.5)	205 (75.9)	1 (50.0)	237 (76.0)	0.3
Neutral	6 (15.0)	48 (17.8)	0 (0)	54 (17.3)	
Disagree/strongly disagree	3 (7.5)	17 (6.3)	1 (50.0)	21 (6.7)	
Helped cope with anxiety					
Agree/strongly agree	29 (72.5)	199 (73.7)	1 (50.0)	229 (73.4)	0.2
Neutral	7 (17.5)	56 (20.7)	0 (0)	63 (20.2)	
Disagree/strongly disagree	4 (10.0)	15 (5.6)	1 (50.0)	20 (6.4)	
Helped cope with depression					
Agree/strongly agree	24 (60.0)	184 (68.1)	1 (50.0)	209 (67.0)	0.2
Neutral	14 (35.0)	67 (24.8)	0 (0)	81 (26.0)	
Disagree/strongly disagree	2 (5.0)	19 (7.0)	1 (50.0)	22 (7.1)	
Helped cope with loneliness					
Agree/strongly agree	19 (47.5)	165 (61.1)	1 (50.0)	185 (59.3)	0.7
Neutral	14 (35.0)	83 (30.7)	0 (0)	97 (31.1)	
Disagree/strongly disagree	7 (17.5)	22 (8.1)	1 (50.0)	30 (9.6)	
Felt connected to a support system					
Agree/strongly agree	31 (77.5)	216 (80.0)	1 (50.0)	248 (79.5)	0.06
Neutral	9 (22.5)	38 (14.1)	0 (0)	47 (15.1)	
Disagree/strongly disagree	0 (0.0)	16 (5.6)	1 (50.0)	17 (5.4)	
Felt hopeful about managing mental health/addiction issues					
Agree/strongly agree	36 (90.0)	206 (76.3)	1 (50.0)	243 (77.9)	0.1
Neutral	3 (7.5)	44 (16.3)	0 (0)	47 (15.1)	
Disagree/strongly disagree	1 (2.5)	20 (7.4)	1 (50.0)	22 (7.1)	
Improved overall mental wellbeing					
Agree/strongly agree	36 (90.0)	207 (76.7)	1 (50.0)	244 (78.2)	0.1
Neutral	3 (7.5)	45 (16.7)	0 (0)	48 (15.4)	
Disagree/strongly disagree	1 (2.5)	18 (6.7)	1 (50.0)	20 (6.4)	
Enhanced quality of life					
Agree/strongly agree	32 (80.0)	194 (71.9)	1 (50.0)	227 (72.8)	0.2
Neutral	7 (17.5)	58 (21.5)	0 (0)	65 (20.8)	
Disagree/strongly disagree	1 (2.5)	18 (6.7)	1 (50.0)	20 (6.4)	

Participants expressed their opinions on the supportive messages they received during the wildfires, as illustrated in Table 3. The majority of participants (68.9%) felt that the messages were positive, always affirmative (65.2%), and relevant (51.9%). About 81% of the participants read the messages and took time to reflect on the messages, 90% always read the messages, 70.6% were very satisfied with the frequency of the daily text; 20% preferred twice daily, and while 73.9% preferred once daily, although not statistically significant. Compared to males and other gender identities, a larger percentage of females indicated that they at least returned to read the messages (79.8%) and always found the text to be succinct (*p* < 0.05).

**Table 3:** Differences in feedback on Text4Hope messages based on gender.

Feedback	Male *n* (%)	Female *n* (%)	Other gender *n* (%)	Total *n* (%)	*p*-value
The messages were positive					
Always	28 (73.7)	185 (68.8)	0 (0.0)	213 (68.9)	0.6
Often	10 (26.3)	72 (26.8)	1 (50.0)	83 (26.9)	
Sometimes	0 (0.0)	9 (3.3)	0 (0.0)	9 (2.9)	
Rarely	0 (0.0)	3 (1.1)	1 (50.0)	4 (1.3)	
The messages were affirmative					
Always	26 (66.7)	175 (65.1)	1 (50.0)	202 (65.2)	0.1
Often	12 (30.8)	70 (26.0)	0 (0.0)	82 (26.5)	
Sometimes	1 (2.6)	19 (7.1)	0 (0.0)	20 (6.5)	
Rarely	0 (0.0)	5 (1.9)	0 (0.0)	5 (1.6)	
Never	0 (0.0)	0 (0.0)	1 (50.0)	1 (0.3)	
The messages were succinct					
Always	22 (56.4)	159 (59.1)	1 (50.0)	182 (58.7)	0.02
Often	14 (35.9)	71 (26.4)	0 (0.0)	85 (27.4)	
Sometimes	2 (5.1)	35 (13.0)	0 (0.0)	37 (11.9)	
Rarely	1 (2.6)	4 (1.5)	0 (0.0)	5 (1.6)	
Never	0 (0.0)	0 (0.0)	1 (50.0)	1 (0.3)	
The messages were relevant					
Always	23 (59.0)	138 (51.3)	0 (0.0)	161 (51.9)	0.1
Often	10 (25.6)	86 (32.0)	0 (0.0)	96 (31.0)	
Sometimes	6 (15.4)	37 (13.8)	1 (50.0)	44 (14.2)	
Rarely	0 (0.0)	8 (3.0)	1 (50.0)	9 (2.9)	
Action taken after reading the messages					
Took no action	2 (5.1)	15 (5.6)	0 (0.0)	17 (5.5)	0.22
Took time to reflect on the messages	32 (82.1)	219 (81.4)	1 (50.0)	252 (81.3)	
Took a positive action	5 (12.8)	32 (11.9)	0 (0.0)	37 (11.9)	
Took a negative action	0 (0.0)	1 (0.4)	1 (50.0)	2 (0.6)	
Never read the messages	0 (0.0)	1 (0.4)	0 (0.0)	1 (0.3)	
Other	0 (0.0)	1 (0.4)	0 (0.0)	1 (0.3)	
Frequency of reading the messages					
Always	37 (94.9)	240 (89.2)	2 (100)	279 (90.0)	0.8
Often	2 (5.1)	25 (9.3)	0 (0.0)	27 (8.7)	
Sometimes	0 (0.0)	4 (1.5)	0 (0.0)	4 (1.3)	
Return to read the messages					
Always	3 (7.7)	18 (6.7)	0 (0.0)	21 (6.8)	0.01
Often	4 (10.3)	77 (28.6)	1 (50.0)	82 (26.5)	
Sometimes	17 (43.6)	127 (47.2)	0 (0.0)	144 (46.5)	
Rarely	10 (25.6)	34 (12.6)	1 (50.0)	45 (14.5)	
Never	5 (12.8)	13 (4.8)	0 (0.0)	18 (5.8)	
Satisfied with the frequency of messages					
Very satisfied	28 (71.8)	191 (71.0)	0 (0.0)	219 (70.6)	0.2
Satisfied	8 (20.5)	66 (24.5)	2 (100)	76 (24.5)	
Mixed	3 (7.7)	12 (4.5)	0 (0)	15 (4.8)	
Preferred frequency to receive message					
Twice daily	11 (28.2)	50 (18.6)	0 (0.0)	61 (19.7)	0.5
Once daily	25 (64.1)	202 (75.1)	2 (100)	229 (73.9)	
Once every other day	3 (7.7)	14 (5.2)	0 (0)	17 (5.5)	
Once weekly	0 (0.0)	3 (1.1)	0 (0)	3 (1.0)	

For the overall sample, the mean satisfaction score was 8.66 (SD = 1.63) out of 10. Table 4 shows a one-way ANOVA comparing the mean satisfaction scores across age and gender. The analysis revealed significant differences in satisfaction scores across different age groups, with an *F*-value of 5.99 (*p* < 0.001). The estimated omega squared (*ω*^2^ = 0.037) for age suggests a medium to large effect size, accounting for 3.7% of the variance in satisfaction scores. Significant differences were also observed between different gender groups, with an *F*-value of 6.91 (*p* = 0.001). The effect size for gender (using partial eta squared, ηp^2^) was 0.04, suggesting a small to moderate effect size and accounting for 4% of the variance in satisfaction scores.

**Table 4:** One-way analysis of variance comparing the mean scores on the satisfaction scale across demographic characteristics.

Demographic characteristic	*N*	Mean (SD)	*F* (ANOVA/Welch)	*p*-value	Effect size
Age (years)					
≤30	29	7.28 (2.58)	5.99^a^	<0.001	0.037
31–50	104	8.48 (1.62)			
51–65	135	9.0 (1.32)			
>65	40	8.95 (1.06)			
Gender^b^					
Male	39	8.79 (1.24)	6.91	0.001	0.04
Female	267	8.67 (1.63)			
Other gender	2	4.50 (3.54)			

a Welch test.

b Conducted among participants with complete overall satisfaction scores. Four participants were excluded because of missing overall satisfaction scores.

Table 5 illustrates the *post hoc* analysis. To investigate specific group differences when variances and sample sizes are unequal, ensuring valid pairwise comparisons, the Games-Howell *post hoc* analysis was used which revealed that participants who were ≤30 years of age had significantly lower satisfaction scores compared to those who were between 51 and 65 years age group inclusive (*M* = 7.28, SD = 2.58) and (*M* = 9.0, SD = 1.32), respectively, with a mean difference of −1.73, SE = 0.49, *p* = 0.007, 95% CI [−3.07, −0.39].

**Table 5:** Tukey and Games-Howell *post hoc*.

Satisfaction scale		Mean difference	Standard error	*p*-value	95% Confidence Interval	
					Lower bound	Upper bound
Age^a^						
*I*	*J*					
≤30	31–50 y	−1.20	0.50	0.09	−2.57	0.16
	51–65 y	−1.73^*^	0.49	0.007	−3.07	−0.39
	>65 y	−1.67^*^	0.51	0.01	−3.04	−0.31
31–50 y	≤30	1.20	0.50	0.09	−0.16	2.57
	51–65 y	−0.53^*^	0.19	0.039	−1.03	−0.02
	>65 y	−0.47	0.23	0.18	−1.07	0.13
51–65 y	≤30	1.73^*^	0.49	0.007	0.39	3.07
	31–50 y	0.53^*^	0.19	0.039	0.02	1.03
	>65 y	0.06	0.20	0.99	−0.48	0.59
>65 y	≤30	1.67^*^	0.51	0.01	0.31	3.04
	31–50 y	0.47	0.23	0.18	−0.13	1.07
	51–65 y	−0.06	0.20	0.99	−0.59	0.48
Gender						
Male	Female	0.12	0.27	0.89	−0.52	0.77
	Other gender	4.29^*^	1.16	<0.001	1.56	7.03
Female	Male	−0.12	0.27	0.89	−0.77	0.52
	Other gender	4.17^*^	1.13	<0.001	1.49	6.84
Other gender	Male	−4.29^*^	1.16	<0.001	−7.03	−1.56
	Female	−4.17^*^	1.13	<0.001	−6.84	−1.49

* Significance at *p* < 0.05 level.

a Games-Howell test was used.

The ≤ 30 years age group also showed significantly lower satisfaction scores compared to the >65 years age group (*M* = 7.28, SD = 2.58) and (*M* = 8.95, SD = 1.06), respectively, with a mean difference of −1.67, SE = 0.51, *p* = 0.01, 95% CI [−3.04, −0.31].

Significantly higher mean scores were observed in the 51–65-year-old age group compared to the 31–50-year-old age group (*M* = 9.0, SD = 1.32) and (*M* = 8.48, SD = 1.62), respectively, with a mean difference of −0.53, SE = 0.19, *p* = 0.039, 95% CI [−1.03, −0.02].

In terms of gender groups, the participants who self-identified as “Other gender” reported significantly lower satisfaction scores (*M* = 4.50, SD = 3.54) compared to the participants who self-identified as “Male” (*M* = 8.79, SD = 1.24), with a mean difference of −4.29, SE = 1.16, *p* < 0.001, 95% CI [−7.03, −1.56].

Similarly, the participants who were self-identified as “Other gender” also reported significantly lower satisfaction scores compared to the participants who were self-identified as “Female” (*M* = 8.67, SD = 1.63) with a mean difference of −4.17, SE = 1.13, *p* < 0.001, 95% CI [−6.84, −1.49].

### Qualitative data

This section of the qualitative study aims to understand how participants perceive and incorporate their experiences with the Text4Hope programs into their world (33, 34). Over 3 months, participants were asked to consider how the text messages they received affected their day-to-day lives and their experiences and recommendations. Two primary themes and six subthemes were identified from the data. Figure 1 illustrates the themes and subthemes.

**Figure 1:**
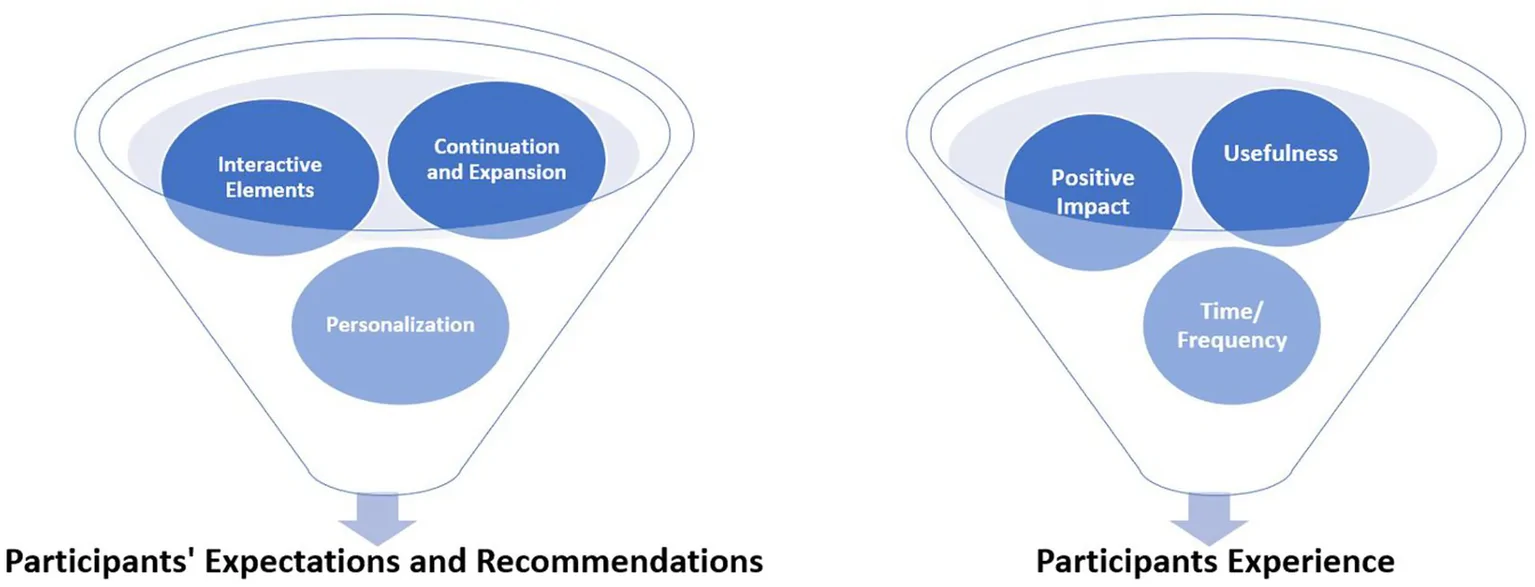
Summary diagram of themes and subthemes.

While some participants reported favorable outcomes from their involvement in the Text4Hope intervention program, a few showed conflicting attitudes about certain elements of the program. A few participants noted that the text messages were somewhat generic at times, and they wished the contents were tailored according to their personal needs. On a similar note, a few participants expressed a desire for the intervention to include interactive elements like videos and pictures. Additionally, some participants also mentioned making adjustments concerning the frequency of sending the messages. These comments highlight opportunities for improving user experience and tailoring future supportive text messaging interventions.

### Participants’ expectations and recommendations

This theme explores participants’ initial expectations and recommendations for improving the Text4Hope-AB and Text4Hope-NS services. It delves into what participants hoped to achieve through the program and the underlying reasons for their suggestions to enhance and extend the service.

#### Continuation and expansion

Participants highly desired the continuation and expansion of the Text4Hope-AB and Text4Hope-NS services. They emphasized the need for ongoing assistance and suggested that prolonging the intervention’s length would enhance its benefits. This desire for sustained and expanded support underscores the importance of the service in their mental health maintenance and overall wellbeing. The participants’ feedback indicates that longer-term engagement with the service could provide even greater advantages, reflecting their recognition of its ongoing relevance and perceived effectiveness. Some participants said,

*“This is all I have. Please find a way to sustain this wonderful service. And thank you to all that have invested in this. You are life savers.”*

*“Please keep Text4hope going. It is making a noticeable difference in a world where no mental and emotional support is available.”*

*“Make it an ongoing and easy to extend service.”*

*“Please keep sending, they are very helpful, Thanks.”*

*“Perhaps it could be an all the time service? Not just for emergency.”*

#### Interactive elements

Another expectation and recommendation was to add additional interactive components to the service. A few participants believed that visuals or links to other resources would make the intervention more interactive and increase their involvement.

*“Add a photo of nice visual imagery. Flowers scenes that can be relaxing to reflect on.”*

*“Add links to support the daily messages.”*

*“I liked it when you included what’s happening currently in province maybe start a multi platform approach so can include beautiful photos and community of Alberta’s pursuing wellness.”*

#### Personalization

Personalization emerged as a key subtheme, with participants expressing a preference for messages that are tailored to their specific needs. They felt that personalized messages would be more impactful, thereby increasing their overall satisfaction with the service. Some participants shared:

*“I think that most people in trouble need more direct, one on one, personal assistance/intervention to make a difference/improvement.”*

*“I think some messages on awareness of feelings or validating feelings would.be supportive. Not all ‘fix it’ type messages.”*

*“Can you add a daily message on grief and loss? I have found these daily messages encouraging and important.”*

*“Perhaps have a different one for depression, a different one for grief etc.”*

### Participants experience

This theme investigates participants’ lived experiences with the Text4Hope services, focusing on the perceived impact and utility of the messages. This theme captures the core experiences and the meaning participants attributed to the support provided, including their desire for continued and expanded services.

#### Usefulness

Participants’ experiences with the Text4Hope services revealed a profound appreciation for the perceived usefulness and guidance provided by the daily texts. The majority of participants reported that they found the service helpful and valuable. Their positive experiences were deeply rooted in their perception of the service’s utility. Participants described it as:

*“I was not impacted significantly by the wildfires but I found the text 4 hope messages helpful.”*

*“Great job, keep up the amazing work. I have been blessed by this service. It helps people especially those that may not have any one to talk to or get messages from. Thank you for this amazing service.”*

*“Having a consistent positive message to start each day help to set the tone for mental health for the day. It is important to keep reinforcing that a person is not alone in their mental health struggles, that it is not abnormal to feel depressed and anxious at times, and that there is support available. The issue is to make those supports known to vulnerable people and to make them easy to access. This daily phone reminder system is quick and easy.”*

*“I felt it complimented my efforts to deal with depression*. *I cannot think of any ways to improve except continue with this program*!”

*“I’ve been so impressed by the Text4Hope-AB that I’ve recommended this program to friends and family………. Having Text4Hope life has made a significant and positive difference in starting my day. Thank you so much for continuing this program! It has made a difference to my day and that of so many others dealing with physical and emotional pain/issues in their daily lives 🙏🏻💙💙💙.”*

#### Positive impact

The Text4Hope services was perceived by participants as supportive and psychologically reassuring. Many participants felt connected to a support system and reflected on the messages and shared with family and friends.

*“Gave me the sense of support that I do not get from family or friends. I love reading encouragement. I looked forward to the txt each day.”*

*“Sad to say this but Text4Hope makes me feel like I have a friend. Most importantly I feel like some one cares.”*

*“I read and reflect and try to introduce it into my life somehow, including when it says things like giving and helping others to improve my mental health.”*

*“I appreciate the positive messages and often pause to reflect on them.”*

*“It is in part because of these messages and reminders that I would remember that I have value. I would share these messages with friends or family. I would post them on my social media but I wasn’t sure I would be allowed. Thank you for supporting me and getting me out of my ‘spin’ it lasted about 5 days but I saw a counsellor and received these messages and took action on the recommendations of my counsellor. (Free of charge)*:*).”*

*“I look forward to the message every day and have shared them with others and they ended up signing up also.”*

#### Time/frequency

Participants had varied opinions about the timing and frequency of the messages. Some appreciated the daily messages as well-timed and appropriately spaced, while others thought otherwise of frequency, wanting more. Some participants described:

*“Twice a day at 9 and 5 would be wonderful or 8 p.m. before bed they are so positive and encouraging.”*

*“Just continue what you are doing, I would like messages twice per day.”*

*“Have the texts twice per day.”*

*“Maybe a reminder about the text or sending it twice a day, same one in the am and then pm.”*

## Discussion

This study contributes to the growing literature on digital mental health interventions by specifically evaluating participant satisfaction, user experience, and perceptions of supportive text messaging interventions delivered during active wildfire emergencies in two Canadian provinces. Unlike previous studies that focused primarily on mental health outcomes, the present study provides insight into the acceptability and perceived usefulness of scalable text-based psychological support during natural disasters.

This study sought to understand Canadians’ experiences with the supportive mental health texting services, Text4Hope-AB and Text4Hope-NS, provided during the peak of the wildfire season in 2023 in Alberta and Nova Scotia. The goals were to examine the general participants’ experience with the Text4Hope-AB and Text4Hope-NS programs and to explore the differences in satisfaction based on age and gender.

The results of this study indicate that the supportive text message intervention was well-received by participants, as reflected by the high mean satisfaction score of 8.66 (SD = 1.63) out of 10. Participants generally reported high satisfaction with the frequency of the messages, with 71.8% of males and 71.0% of females agreeing they were satisfied or very satisfied. Other gender participants reported 100% satisfaction, though the sample size for this group was small. This suggests that, overall, participants were satisfied with the messages. The relatively high score implies that satisfaction was consistently positive across the majority of participants. A similar study providing a supportive text intervention showed an overall mean score of program satisfaction of 85.12 out of 100 (16). Another survey conducted during the COVID-19 pandemic recorded an 8.55 (SD 1.78) overall mean satisfaction score out of 10 (31). These high satisfaction levels indicate that participants generally perceived the text messages supportive and beneficial.

Findings from the study revealed that the older participants were overall more satisfied with the texting services than the younger age group of participants, and the “Male” and “Female” participants were significantly more satisfied with the provided texting service than the participants who self-identified as “Other gender.” Although statistically significant differences in satisfaction were observed across some demographic groups, the associated effect sizes were relatively small, indicating that these demographic factors explained only a limited proportion of the variance in satisfaction scores. Therefore, the practical significance of these differences should be interpreted cautiously.

The age differences in satisfaction scores with the text message intervention align with existing literature, which often points out that older adults may derive more benefit and satisfaction from health interventions due to their higher healthcare needs and possibly lower familiarity with more interactive digital platforms compared to younger adults (35–37). Older adults tend to appreciate the simplicity and directness of text-based interventions, which may explain the higher satisfaction scores observed in our study (38). Other research indicates that satisfaction with digital health interventions tends to increase with age (39, 40). Another study reported that older adults generally reported higher satisfaction with health-related technology, possibly due to their greater need for health monitoring and support during wildfires (41). Additionally, older adults often appreciate the convenience and accessibility of technology-based health interventions, which may contribute to higher satisfaction levels (42).

Conversely, younger adults may be less satisfied with text message interventions. Younger adults, on the other hand, might prefer more interactive and engaging platforms (43), such as mobile apps, that offer richer features and interactivity compared to simple text message interventions (44–46). This discrepancy highlights the need for further research to understand the nuanced relationship between age and satisfaction with digital health interventions.

Although statistical differences in satisfaction scores were observed across gender groups, these findings should be interpreted with caution because only two participants identified as “other gender.” The very small subgroup size limits the reliability, stability, and generalizability of these findings. Therefore, meaningful conclusions regarding satisfaction among gender-diverse participants cannot be drawn from the present study. Some research indicates that gender-diverse individuals face significant challenges, including discrimination and social stigma, which can negatively impact their wellbeing, diminished sense of support, and lower satisfaction with interventions designed to provide emotional and mental health support (47, 48). On the contrary, other studies found that gender-diverse individuals reported higher levels of wellbeing and satisfaction when they experienced social and legal recognition (25). This suggests that support and acceptance from the community can significantly enhance satisfaction levels among gender-diverse individuals. Similarly, Budge et al. emphasized the role of social support and resilience in the wellbeing of transgender individuals (26).

### Participants’ expectations and recommendations versus participants’ experience

Participants reported that the Text4Hope-AB and Text4Hope-NS programs were useful, appreciating the daily messages’ practical advice and emotional support. Most participants generally felt the messages were positive, succinct, relevant, and affirmative. This highlights the perceived usefulness of text message interventions in providing support during wildfires. This finding is supported by a study proving health intervention through short-message service (SMS), which demonstrated that text messaging interventions could promote health behavior change and provide valuable support (49). Even though a few participants stated that they had positive experiences with the intervention, the results from the study showed some areas where improvements were needed. A few participants thought that the messages were too general in nature and they would like more customization, interactivity, and flexibility in the way messages were conveyed. The results show that, whereas the use of standardized text messages is acceptable among many people during disasters, there may be a need to tailor messages more for the benefit of recipients. Mental health interventions delivered through digital or text-based platforms often resonate well with participants, particularly when the tone is positive and affirming (50). The relevance of supportive text messages is crucial in maintaining user engagement with mental health interventions, particularly when the content is personalized to the participant’s current emotional or mental state (51). The perceived usefulness of the service was a significant factor in participants’ overall positive experience. Various studies have recorded the positive impact of text messaging intervention, especially during traumatic events, with 75.6–84% of participants reporting improved overall mental wellbeing (16, 31). In our study, more than 60% were able to cope with stress, anxiety, and depression with the supportive messages they received during the wildfire. In comparison, approximately 80% were hopeful they could manage other mental health and addiction services. These results may explain why participants expressed satisfaction with the program.

Many participants reported feeling more supported and connected to a support system during the wildfires due to the regular receipt of the messages. This finding corresponds with previous research reporting that regular, supportive intervention and communications can enhance feelings of connectedness and emotional wellbeing (52, 53). Moreover, daily supportive messages have been shown to minimize feelings of isolation and improve mental health outcomes, particularly in populations at risk of social isolation (54). Data from our study revealed that 79.5% of participants felt connected to a support system with the daily messages they receive, which aligns with other studies. In a study among participants who have experienced traumatic events and received supportive text intervention, 84% felt connected to a support system, while 75% read the messages and reflected on them (16). Participants also reported sharing text messages with close friends and family, knowing the intervention would positively impact their wellbeing (55).

Participants expressed varied opinions regarding the timing and frequency of the messages. While some appreciated receiving daily messages, viewing them as well-timed and appropriately spaced, others felt that the frequency could be better tailored to their schedules and preferences. Quantitative data showed that most males (64.1%) and females (75.1%) preferred receiving messages once daily, suggesting that daily messaging is optimal for most participants. This is consistent with studies indicating that daily engagement with mental health interventions can improve outcomes through consistent reinforcement (56, 57). However, feedback from the qualitative survey also revealed that some participants preferred receiving messages twice daily instead of once, indicating an opportunity for improvement by customizing the timing and frequency to meet individual needs better. The timing and frequency of message delivery often influence the effectiveness of digital health interventions (55, 58). Tailoring these aspects could enhance user engagement and satisfaction, potentially improving outcomes (59). Additionally, participants’ desire for more frequent messages may imply that the service is perceived as effective, as individuals are more likely to continue with an intervention that they find beneficial (60, 61).

Results from this study highlight that participants strongly desired the program to continue and expand. They emphasized the value of ongoing support, suggesting that extending the duration of the intervention could further enhance its benefits. This demand for expansion indicates that participants found the service beneficial and believe it could reach and assist a broader audience. This recommendation aligns with studies suggesting that sustained interventions are more effective in promoting long-term behavior change and maintaining mental health benefits, instilling optimism in the audience about the potential impact of the intervention (49, 62).

Another key recommendation from participants was the inclusion of more interactive elements in the Text4Hope-AB and Text4Hope-NS services. Many participants felt adding features like pictures or links to additional resources would increase their engagement. The desire for interactivity suggests that some participants prefer to seek a more dynamic and engaging experience, potentially enhancing their satisfaction and adherence to the intervention. This aligns with studies showing that interactive digital health interventions are more engaging (49, 63, 64).

Personalization of text messages emerged as a crucial subtheme, with participants expressing a preference for messages tailored to their specific needs and circumstances. Although the messages were tailored to support individuals during the 2023 wildfires in Alberta and Nova Scotia, participants wanted the messages to be more tailored to their personal mental health needs. They felt that personalized messages would be more relevant and impactful, increasing their overall satisfaction with the service. The importance of personalization is well-documented in the literature, where tailored interventions have been shown to improve engagement and outcomes by addressing users’ specific needs and preferences (49, 65, 66).

### Strengths and limitations

Despite the valuable insights provided by this study, a few limitations must be acknowledged. The study did not include a control group, making it challenging to ascertain whether the increased satisfaction can be directly and solely attributed to the Text4Hope-AB and Text4Hope-NS services. Although the questionnaire used in the study was designed based on relevant literature, it was not a validated instrument. The study’s sample size, though adequate for initial analysis, limits the generalizability of the findings. Also, only two participants identified as “other gender,” limiting the reliability of statistical comparisons involving this subgroup. As a result, gender-related findings should be interpreted cautiously and require confirmation in studies with larger and more diverse samples. Participants were primarily subscribers from a specific demographic area, which may not represent the broader population. Again, the study utilized a self-selected convenience sample of subscribers who voluntarily enrolled in the Text4Hope programs. Although 90% of participants reported always reading the messages, this was based on self-report and could not be independently verified. Future studies should incorporate objective measures of message engagement and comprehension to more accurately assess participants’ exposure to and interaction with supportive text messages over time. Consequently, participants may have been predisposed to view the intervention positively, introducing potential selection and response biases. Furthermore, the subgroup analyses were based on satisfaction ratings rather than validated measures of mental health outcomes. Although these analyses provide insight into the acceptability of the intervention among different demographic groups, they offer limited information regarding differential clinical benefits and should therefore be interpreted as exploratory findings. Additionally, participants could opt out of the Text4Hope intervention at any time; however, data on opt-outs and the complete linkage between message exposure and survey completion were not systematically captured, which introduced potential survivorship bias and limited the assessment of engagement among non-responders. Lastly, the qualitative data were collected through written responses to questions rather than interviews. While this method can reduce interviewer bias and allow participants to reflect on their answers, it may limit the depth and richness of the data compared to face-to-face or telephone interviews. Written responses might not capture the nuances of verbal communication, such as tone, hesitation, or non-verbal cues. Future studies might combine written responses with interviews for a more comprehensive understanding. This study provides valuable insights into participants’ experiences with scalable supportive text messaging interventions (Text4Hope-AB and Text4Hope-NS) during active wildfire disasters, despite its limitations. These findings offer initial evidence of the perceived usefulness and acceptability of low-cost digital mental health services in disaster scenarios.

## Conclusion

Our study underscores the perceived usefulness of the Text4Hope-AB and Text4Hope-NS text message interventions in providing crucial psychological support during wildfires. The significant age differences in satisfaction levels highlight the need for tailored communication strategies that address the diverse needs of different demographic groups. Additionally, the higher satisfaction reported by males and females indicates the importance of inclusive and responsive design in intervention programs. Participants generally reported satisfaction with the Text4Hope-AB and Text4Hope-NS programs, and participant feedback describes the interventions as supportive and psychologically reassuring. The positive feedback could include the novelty of receiving supportive messages during a crisis, which might have temporarily boosted participants’ morale. Long-term studies would be needed to assess the sustained impact of such interventions. Future studies are recommended to explore how similar interventions can be optimized to meet the needs of various populations and other natural disasters. Overall, Text4Hope-AB and Text4Hope-NS represent potentially promising tools for psychological support, offering valuable insights for developing future crisis communication strategies.

## Data Availability

The original contributions presented in the study are included in the article/supplementary material, further inquiries can be directed to the corresponding author.
